# First detection of canine parvovirus type 2b from diarrheic dogs in Himachal Pradesh

**DOI:** 10.14202/vetworld.2016.964-969

**Published:** 2016-09-12

**Authors:** Shalini Sharma, Prasenjit Dhar, Aneesh Thakur, Vivek Sharma, Mandeep Sharma

**Affiliations:** 1 Department of Veterinary Microbiology, Dr. G. C. Negi College of Veterinary and Animal Sciences, Chaudhary Sarwan Kumar Himachal Pradesh Krishi Vishvavidyalaya, Palampur - 176 062, Himachal Pradesh, India; 2 Department of Microbiology, College of Basic Sciences, Chaudhary Sarwan Kumar Himachal Pradesh Krishi Vishvavidyalaya, Palampur - 176 062, Himachal Pradesh, India

**Keywords:** canine parvovirus, molecular typing, phylogenetic analysis, sequencing

## Abstract

**Aim:**

The present study was conducted to detect the presence of canine parvovirus (CPV) among diarrheic dogs in Himachal Pradesh and to identify the most prevalent antigenic variant of CPV based on molecular typing and sequence analysis of VP2 gene.

**Materials and Methods:**

A total of 102 fecal samples were collected from clinical cases of diarrhea or hemorrhagic gastroenteritis from CPV vaccinated or non-vaccinated dogs. Samples were tested using CPV-specific polymerase chain reaction (PCR) targeting VP2 gene, multiplex PCR for detection of CPV-2a and CPV-2b antigenic variants, and a PCR for the detection of CPV-2c. CPV-2b isolate was cultured on Madin-Darby canine kidney (MDCK) cell lines and sequenced using VP2 structural protein gene. Multiple alignment and phylogenetic analysis was done using ClustalW and MEGA6 and inferred using the Neighbor-Joining method.

**Results:**

No sample was found positive for the original CPV strain usually present in the vaccine. However, about 50% (52 out of 102) of the samples were found to be positive with CPV-2ab PCR assay that detects newer variants of CPV circulating in the field. In addition, multiplex PCR assay that identifies both CPV-2ab and CPV-2b revealed that CPV-2b was the major antigenic variant present in the affected dogs. A PCR positive isolate of CPV-2b was adapted to grow in MDCK cells and produced characteristic cytopathic effect after 5^th^ passage. Multiple sequence alignment of VP2 structural gene of CPV-2b isolate (Accession number HG004610) used in the study was found to be similar to other sequenced isolates in NCBI sequence database and showed 98-99% homology.

**Conclusion:**

This study reports the first detection of CPV-2b in dogs with hemorrhagic gastroenteritis in Himachal Pradesh and absence of other antigenic types of CPV. Further, CPV-specific PCR assay can be used for rapid confirmation of circulating virus strains under field conditions.

## Introduction

Canine parvoviral infection is caused by canine parvovirus 2 (CPV-2) of family Parvoviridae. CPV-2 is a non-enveloped single-stranded DNA virus, 5.2 kb in size, has icosahedral symmetry and encodes for two structural (capsid) proteins (VP1 and VP2) and two non-structural proteins (NS1 and NS2). The disease caused by CPV-2 is clinically characterized by severe vomiting and diarrhea leading to fatal dehydration and myocarditis in pups below 3 months of age [1] and is a major cause of mortality in young dogs [2]. In nature, CPV-2 continues to evolve and successful new strains of CPV have an extended host range [3]. The virus is distributed worldwide, and antigenic variation among CPV-2 isolates has been used to further classify the virus into three antigenic variants, namely, CPV-2a, CPV-2b, and CPV-2c that differ in their amino acid sequence and VP2 gene structure [4]. CPV-2 was first isolated in India in 1982 [5] followed by many outbreaks due to CPV-2a [6] and CPV-2b [7]. CPV-2c was first reported in India in 2010 [8].

The original CPV-2 strain is still used for the development of vaccine but has now gradually replaced by new CPV-2a and CPV-2b variants under field conditions in different countries [6,9,10]. The emergence of new pathogenic variants of CPV has led to a genuine concern about the existing vaccines that may not be able to provide adequate protection to the canine population in the future. Another area of increased concern is the heightened virulence of the CPV-2 variants leading to higher shedding in the feces of infected dogs, less number of infectious virus particles to initiate an infection and consequently a more severe disease [11]. The identification of variants of CPV-2 that is currently circulating in the canine population is essential for the understanding of viral evolution and epidemiology as well as for the development of adequate control measures [12].

No information is yet available about the status of CPV infection in dogs in the state of Himachal Pradesh in Northwestern Himalayas in India. Therefore, the present study was conducted to detect the presence of CPV and the circulating antigenic variants among diarrheic dogs in Himachal Pradesh.

## Materials and Methods

### Ethical approval

The study was conducted following due approval by the Institutional Animal Ethics Committee of Dr. G. C. Negi College of Veterinary and Animal Sciences, Palampur, Himachal Pradesh.

### Collection and processing of fecal samples

Fecal samples (n=102) were collected from the rectum of dogs suffering from hemorrhagic gastroenteritis or diarrhea from different regions of Himachal Pradesh. 96 of 102 fecal samples originated from CPV non-vaccinated dogs, whereas 6 fecal samples were from CPV vaccinated dogs. The fecal samples were emulsified in 0.1 M phosphate-buffered saline (PBS) (pH 7.4) containing antibiotic-antimycotic solution (10,000 units penicillin, 10 mg streptomycin, and 25 μg amphotericin B per ml in 0.9% normal saline) (Sigma-Aldrich). This emulsion was then centrifuged at 6000 rpm for 15 min at 4°C. The supernatant was collected, filtered through 0.45 μm sterile filter and was stored at −20°C in two aliquots of 2 ml each. One aliquot was used for molecular studies, whereas another aliquot was used as live viral inoculum for growth in cell culture. A live CPV virus (freeze dried) kindly provided by the Central Military Veterinary Laboratory (CMVL), Meerut, India, was used as positive control virus in cell culture, whereas positive CPV-DNA provided by the School of Animal Biotechnology, Guru Angad Dev Veterinary and Animal Sciences University (GADVASU), Ludhiana, Punjab, India, was used as a positive control in CPV-specific polymerase chain reaction (PCR). DNA obtained from feces of a healthy dog was taken as a negative control in PCR.

### Molecular detection of CPV

#### Extraction of DNA and quantification

DNA was extracted from feces using conventional phenol-chloroform DNA extraction method [13]. The DNA concentration was measured in a NanoDrop ND 1000 spectrophotometer. Approximately, 100 ng of DNA was used for each PCR reaction.

### Polymerase chain reaction for detection of CPV-2

PCR primer sequences used in the study were chosen from published nucleotide sequences of CPV-2, CPV-2a, and CPV-2b [14,15]. The sequences were selected from variable regions of the VP1/VP2 capsid gene. For detection of the original strain of CPV-2, CPV-2 primers were used having a primer sequence of CPV-2 F-5’ GAAGAGTGGTTGTAAATAATA 3’ and CPV-2 R- 5’ CCTATATCACCAAAGTTAGTAG 3’ at location 3025-3045 and 3685-3706, respectively. This primer pair amplifies a 681 bp fragment of the original strain of CPV-2. The PCR program was as follows: 5 min at 94°C, 30 cycles of 30 s at 94°C, 2 min 55°C, followed by 2 min at 72°C and 5 min at 72°C (Eppendorf Mastercycler, Germany).

To detect the new antigenic variants of CPV, a 681 bp fragment encoding capsid protein VP2 of both antigenic types CPV-2a and CPV-2b were amplified from the template DNA using primers CPV-2ab F-5’ GAAGAGTGGTTGTAAATAATT 3’ and CPV-2ab R-5’ CCTATATAACCAAAGTTAGTAC 3’ [16] under similar PCR conditions.

The primer pairs CPV-2 and CPV-2ab that recognize the original CPV type and newer antigenic variants, respectively, were selected from overlapping positions, due to which the difference in nucleotide sequence between the primers is restricted to one base at the 3-end of each primer. As the PCR products were to be the same size upon amplification, the primers were, therefore, used in separate sets of reactions. The PCR reaction was performed as described earlier [17]. The PCR products were electrophoresed along with a 100 bp DNA ladder in 1% agarose gel containing 0.5 μg/ml ethidium bromide and progress of the mobility was monitored by migration of dye.

### Multiplex PCR for detection of CPV-2 antigenic variants

A multiplex PCR was performed for simultaneous detection of CPV-2a and CPV-2b types. All the samples that were positive by CPV-2ab PCR assay were further amplified by a multiplex PCR utilizing primer pairs CPV-2ab-F and CPV-2ab-R amplifying a 681 bp fragment and primers CPV-2b F-5’ CTTTAACCTTCCTGTAACAG 3’ and CPV-2b R-5’ CATAGTTAAATTGGTTATCTAC 3’ amplifying a 427 bp fragment of the gene encoding the capsid protein VP2 [17]. The samples were subjected to multiplex PCR following the similar protocol as described above for primer pair CPV-2ab.

### PCR for detection of CPV-2c antigenic variant

All the fecal samples found negative by PCR with CPV-2, CPV-2ab, and CPV-2b primer pairs were again subjected to another PCR using primer pairs CPV 555 F-5’ AGGAAGATATCCAGAAGGA 3’ and CPV 555 R-5’ GGTGCTAGTTGATATGTAATAAACA 3’ that amplifies a 583 bp fragment of the gene encoding capsid protein VP2 of CPV-2a, CPV-2b, and also CPV-2c types [18]. The PCR product was digested with enzyme *MboII* that selectively recognizes the restriction site “GAAGA” (nucleotide 4062-4066 of the VP2 encoding gene) unique to CPV-2c generating two fragments of 500 and 83 bp. After digestion at 37°C for 2 h and enzyme inactivation at 65°C for 5 min, the digested product was analyzed in 2.5% agarose gel. The PCR program was as follows: 5 min at 94°C, 40 cycles of 30 s at 94°C, 1 min 50°C, followed by 1 min at 72°C and 10 min at 72°C (Eppendorf Master Cycler, Germany).

### Isolation of CPV in cell culture

For isolation of CPV in living cells, three representative virus isolates of antigenic variants were grown in Madin-Darby canine kidney (MDCK) cell line. The MDCK cell line was grown to confluence in Minimal Essential Medium (MEM) (Sigma-Aldrich) containing 10% fetal calf serum (Sigma-Aldrich) at 37°C with 5% CO_2_. When the monolayers were 80-90% confluent, the growth medium was decanted, and 0.1 ml of viral inoculum was added to a 25 cm^2^ tissue culture flask. Coverslip cultures were also prepared for infection and controls. Simultaneous inoculations of similar flasks with an equal volume of sterile PBS served as negative culture controls, whereas the live virus procured from CMVL, Meerut, India, was used as positive culture control. Maintenance of cell cultures was done using MEM with 1% fetal calf serum. The inoculum was allowed to adsorb at 37°C for 1 h. After 1 h, the inoculum was pipetted out, and the monolayer was washed with PBS. Finally, MEM was added to each monolayer including the controls, incubated at 37°C and the monolayer was examined daily for the appearance of cytopathic effects (CPE). The virus infectivity titer of the isolate adapted to cell culture was measured after 7^th^ passage level as reported earlier [19].

### Sequencing of VP2 structural protein gene

For sequencing of VP2 structural protein gene, DNA was extracted from the CPV isolate that adapted well to MDCK cell culture. CPV-specific PCR was performed, and the PCR product was purified using HiPurA™ PCR Product Purification Spin kit (HiMedia). Approximately, 45 μl of DNA containing a total concentration of 1-2 μg was used for partial sequencing of VP2 structural protein gene through a commercial partner.

### Sequence analysis

The obtained CPV-2b sequence was aligned with established sequences of CPV from NCBI database using Basic Local Alignment Search Tool [20]. The sequence was analyzed using Bioedit, and the similarity search was performed based on the available reference sequence. The sequence was submitted to NCBI GenBank for accession number. Multiple alignment and phylogenetic analysis was done using ClustalW and MEGA6 [21] and inferred using the Neighbor-Joining method [22]. The tree is drawn to scale, with branch lengths (0.03236613) in the same units as those of the evolutionary distances used to infer the phylogenetic tree.

## Results and Discussion

**Figure 1:**
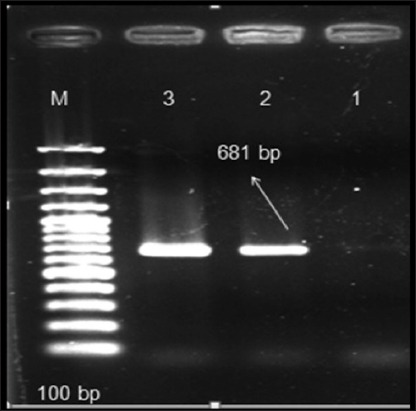
Canine parvovirus 2ab polymerase chain reaction (PCR) assay amplifies VP2 gene and generates a 681 bp PCR product. Lane M represents ladder, sample 1 is negative control, sample 2 is positive control, and sample 3 is positive fecal sample.

**Figure 2:**
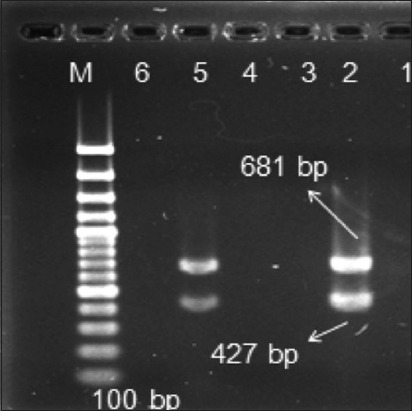
Canine parvovirus (CPV)-2ab/CPV2b multiplex polymerase chain reaction (PCR) assay simultaneously detects CPV-2a and CPV-2b antigen types. The multiplex assay generates 681 and 427 bp PCR products with CPV-2b antigenic strain. Lane M represents ladder, sample 1 is negative control, sample 2 is positive sample, sample 5 is positive fecal sample, and 3, 4, and 6 are negative samples.

This is the first study reporting the role of CPV-2 infection in dogs in the State of Himachal Pradesh in Northwestern Himalayas in India and the detection of a particular variant in causation of disease. A total of 102 fecal samples were collected from dogs with diarrhea or hemorrhagic enteritis. 96, of these 102 fecal samples, were collected from dogs that were not vaccinated against CPV based on the clinical history while 6 dogs were vaccinated. All the 102 samples analyzed by CPV-2 PCR assay were found to be negative for the presence of an original strain of CPV. However, CPV-2ab PCR assay revealed that almost 50% (52 out of 102) of the fecal samples were positive by VP2 gene based PCR with an expected 681 bp product amplicon size (Figure-1). All of these 52 positive samples originated from the 96 diarrheic dogs that were not vaccinated against CPV. CPV-2ab/CPV-2b multiplex PCR assay further confirmed these results as all the 52 positive samples were found to harbor only CPV-2b type antigenic variant (Figure-2). In addition, no sample could be amplified by CPV 555 primer pairs (data not shown), which meant that CPV-2c strain was absent from the samples collected in this study.

As none of the samples were positive by CPV-2 PCR assay, it can be assumed that the original strain of CPV that is normally incorporated into CPV vaccines no longer circulates in the local canine population of the state. The presence of the original strain of CPV in commercially available vaccines and absence of this strain from the field is an area of deep concern. No information is available if the vaccinated animals are adequately protected against newer antigenic types using available CPV vaccines. However, the original strain has recently been isolated from domestic dog populations of many countries [23,24]. In India, where no thorough investigation has been conducted to study the countrywide prevalence of CPV, we cannot rule out the fact that similar cases in India may have gone undetected. Whether the original strain has truly disappeared from the country can only be addressed by a Pan-Indian study involving a large number of samples from different geographical locations over a period of few years.

**Figure 3:**
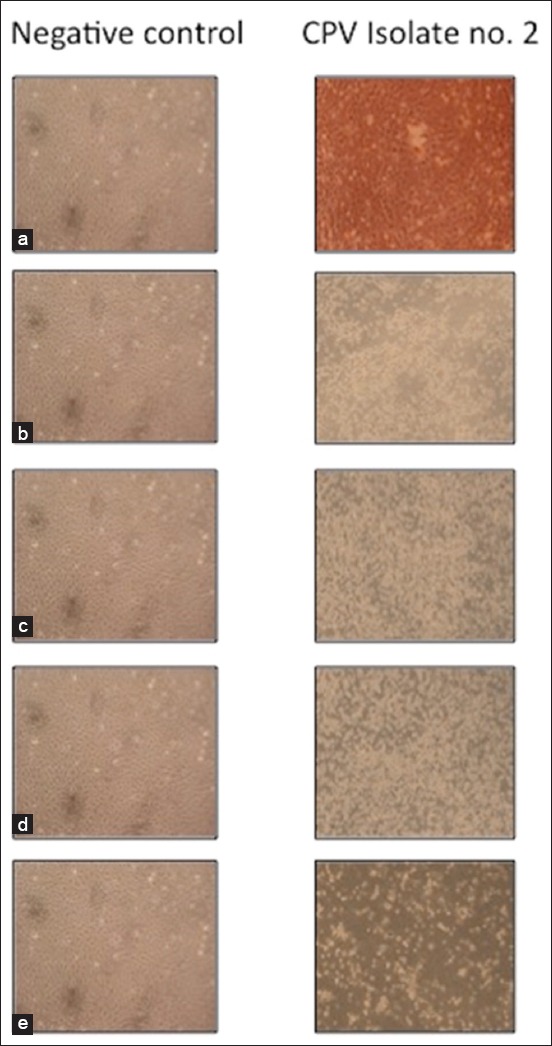
Cytopathic effects produced by canine parvovirus on Madin-Darby canine kidney cell culture at different time points (100×), (a) Cell monolayer after 24 h showing no visible change, (b) cell monolayer after 48 h showing slight rounding of cells, (c) cell monolayer after 72 h showing rounding of cells, (d) cell monolayer after 96 h showing rounding and sloughing of cells, (e) cell monolayer after 120 h showing complete sloughing of cells.

An isolate of CPV-2b antigenic variant confirmed in this study was then cultured on MDCK cell lines and subjected to phylogenetic analysis by sequencing of VP-2 encoding gene (Figure-3). On MDCK cell lines, the CPV isolate could be adapted with characteristic CPE only after 5^th^ passage (Table-1). Low frequency of adaptation of field isolates of CPV in homologous cell culture like-MDCK could be because, in a natural infection, the predilection site is usually the intestine, whereas MDCK cells are usually made up of cells of renal origin. Another reason could be that CPV requires cells in rapidly dividing mitotic phase for initiating infection which probably was not fulfilled with renal cells. There might be other stimulating factors from the host that are present *in vivo* but absent under *in vitro* conditions. However, CPE at the 7^th^ passage level was characterized by rounding and shortening of cells that started 48 h post infection (p.i.) and was followed by slight detachment at 72 h p.i and complete detachment of the monolayer at 120 h p.i as has been reported earlier [25]. The positive virus control started to show CPE at 3^rd^ passage level, whereas no detectable changes were seen with the negative control sample. The virus infectivity titer of the isolate measured after 7^th^ passage level was found to be 6.6 × 10^4^ TCID_50_/ml.

**Table-1:** Details of CPE on the MDCK cells at different passage levels.

Passage level	CPV isolate no. 2				

	24 h p.i	48 h p.i	72 h p.i	96 h p.i	120 h p.i
1^st^	-	-	-	-	-
2^nd^	-	-	-	-	-
3^rd^	-	-	-	-	-
4^th^	-	-	-	-	-
5^th^	-	-	-	-	+
6^th^	-	-	+	+	+
7^th^	-	+	+	++	+++

-: No visible change in cell monolayer, +: Slight rounding, ++: Rounding and slight sloughing/detachment of cell monolayer, +++: Complete sloughing/detachment of cell monolayer, CPE: Cytopathic effects, MDCK: Madin-Darby canine kidney, p.i.: Post infection

**Figure 4:**
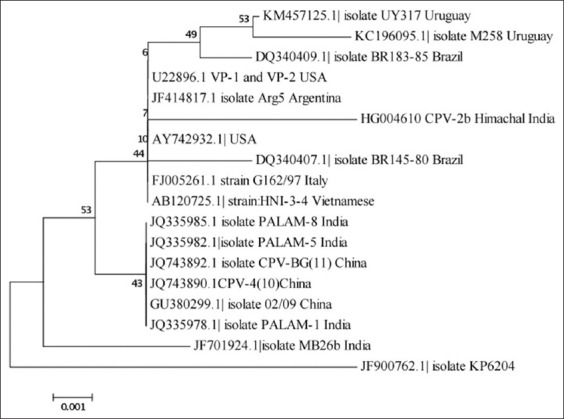
Phylogenetic tree constructed using canine parvovirus sequence under study ([CPV-2b], HG004610) and reference CPV-2b sequences, KM457125.1 (UY317, Uruguay), KC196095.1 (M258, Uruguay), DQ340409.1 (BR-183-85, Brazil), U22896.1 (VP-1 and VP-2, USA), JF414817.1 (Arg5, Argentina), AY742932.1 (USA), DQ340407.1 (BR145-80, Brazil), FJ005261.1 (G162/97, Italy), AB120725.1 (HNI-3-4, Vietnam), JQ335985.1 (PALAM-8, India), JQ335982.1 (PALAM-5, India), JQ743892.1 (CPV-BG [11], China), JQ743890.1 (CPV-4 [10], China), GU380299.1 (02/09, China), JQ335978.1 (PALAM-1, India), JF701924.1 (MB26b, India), and JF900762.1 (KP6204, India).

Multiple sequence alignment of VP2 structural gene of the CPV-2b isolate (Accession number HG004610) used in the study was found to be similar to other sequenced isolates and showed 98-99% homology. The nucleotide sequence data can further be used to know the percent homology and for phylogenetic analysis of CPV-2 isolates from different geographical regions [11,26]. It can also be used to differentiate CPV-2a and CPV-2b types from the original CPV-2 type [5], field CPV strains from vaccine strain [12], and to identify CPV-2c variants [12,27]. Phylogenetic analyses of the VP2 structural gene of CPV isolate in the study revealed its proximity to CPV isolates originating from Argentina and USA though our isolate formed its own cluster (Figure-4). Other CPV isolates from India and China were clustered in a separate group. The results of the current study could not explain the proximity of our isolate with the American isolates and needs further investigations.

## Conclusion

We have shown the presence of CPV infection in the non-vaccinated canine population of the state of Himachal Pradesh in Northwestern Himalayas in India caused by a single antigenic variant, CPV-2b. No other antigenic types of CPV could be recovered in our study. The results suggest the usefulness of CPV PCR assay for rapid confirmation of viral strain under field conditions. Overall, our findings suggest that vaccinated dogs are still protected against CPV infection. However, the presence of CPV-2b antigenic variant in the field, which is not present in currently available vaccines, can lead to vaccine failure and subsequent parvoviral disease in future. Therefore, new strain should be incorporated in presently available vaccines for the improved control of CPV infection, and a continuous monitoring should be carried out to record emerging strains in the field.

## Authors’ Contributions

PD conceived and designed the study. SS performed the experiments. AT analyzed the data, wrote and revised the manuscript. VS analyzed sequencing data and corrected the manuscript. MS contributed to manuscript correction and supervision of the study. All authors read and approved the final manuscript.
